# Emergency Medicine Procedures: A Review of Approved Instructional Resources from the World of Free Open Access Medical Education

**DOI:** 10.7759/cureus.45986

**Published:** 2023-09-26

**Authors:** Jay Khadpe, Christopher N Belcher, Katrina Damore, Jessica Hoglund, Linda Katirji, Matthew Melton, Andrew Grock

**Affiliations:** 1 Department of Emergency Medicine, University of Florida College of Medicine - Jacksonville, Jacksonville, USA; 2 Department of Emergency Medicine, University of Kentucky, Lexington, USA; 3 Department of Emergency Medicine, New York City Health & Hospitals - South Brooklyn Health, Brooklyn, USA; 4 Department of Emergency Medicine, Atrium Health, Charlotte, USA; 5 Emergency Department, VA Greater Los Angeles Healthcare System, Los Angeles, USA; 6 Department of Emergency Medicine, University of California, Los Angeles, USA

**Keywords:** lumbar puncture (lp), wound closure, laryngoscopy, regional nerve blocks, regional anesthesia, medical education, procedures, free open access medical education, emergency medicine

## Abstract

The Academic Life in Emergency Medicine (ALiEM) Approved Instructional Resources (AIR) Series was created in 2014 to address the Free Open Access Medical Education (FOAM) movement’s decentralized nature and lack of inherent peer review. The AIR series provides a topic-based, curated list of online educational content vetted by academic emergency medicine (EM) faculty that meets individualized interactive instruction criteria for EM trainees. Relevant FOAM resources were identified from the top 50 FOAM websites using the Social Media Index and then scored by EM faculty using a validated instrument to identify the highest quality posts related to a topic. This article reviews FOAM resources pertaining to EM procedures that were labeled as an "Approved Instructional Resource" or "Honorable Mention" using the AIR series methodology.

## Introduction and background

Since the term was coined in 2012, Free Open Access Medical Education (FOAM) has become a fundamental component in the delivery of graduate medical education [1]. The meteoric rise of the FOAM movement carries with it the challenges of decentralized resources, no traditional peer review process, and a lack of curricular comprehensiveness [2]. The Academic Life in Emergency Medicine (ALiEM) Approved Instructional Resources (AIR) series was founded to address these challenges by providing a centralized, curated list of relevant, high-quality FOAM resources for emergency medicine (EM) trainees. This article reviews FOAM resources on EM procedures identified as either “Approved Instructional Resource” or “Honorable Mention” through the AIR series methodology [3].

Members of the ALiEM AIR Executive Board have previously published reviews of FOAM identified by the AIR series on a variety of EM core content topics [4-14]. These reviews help to facilitate the dissemination of vetted, high-quality FOAM resources to the EM community within existing published literature. The next section of this article discusses the process to identify FOAM resources pertaining to EM procedures rated at the level of either "Approved Instructional Resource" or "Honorable Mention" by the AIR series in January 2023.

## Review

Methods

**Figure 1:**
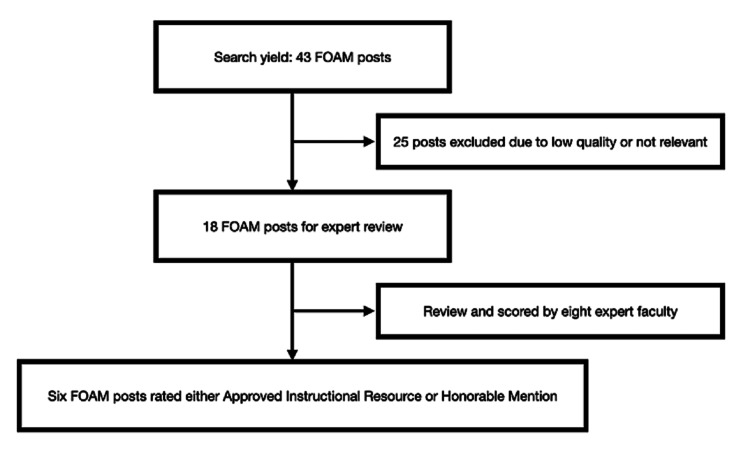
FOAM posts search and review results FOAM: Free Open Access Medical Education

The AIR series first identified the highest-quality resources regarding EM procedures through a search for relevant resources published by the top 50 FOAM websites (as identified by the Social Media Index (SMi) within the past year (January 2022 to January 2023). The SMi was designed to be the FOAM corollary to the journal impact factor. Derived from three components: Alexa rank, Twitter followers, and Facebook likes [15], SMi has been shown to correlate moderately to strongly with other evaluation tools for quality [16]. As the Alexa rank was discontinued in 2022, the SMi was last updated at the end of 2021 and will be replaced with the Digital Impact Factor in future iterations of the AIR series [17]. Search terms were identified using the Model of the Clinical Practice of Emergency Medicine published by the American Board of Emergency Medicine under the heading of “Procedures and Skills Integral to the Practice of Emergency Medicine” [18]. The search terms were then utilized on each of the websites to identify relevant posts. If no search function was available for a particular website, posts published during the assigned timeframe were individually reviewed for relevance to procedures in EM. Posts were considered low quality or not relevant and therefore excluded if not published within the designated timeframe, authors were not cited, or the post was not within the content area of focus. Figure 1 displays the search results to identify those FOAM posts that were selected and reviewed for the AIR Series module on EM procedures in January 2023.

From those FOAM posts identified as relevant, each resource then underwent review by eight experts in EM education from the ALiEM AIR Executive Board that consists of more than 18 EM faculty from academic training programs across the United States. Board members recused themselves from the evaluation of posts that they had any role in authoring, editing, or publishing. Posts were reviewed and assigned a numeric score using the AIR scoring tool, consisting of five measurement outcomes, each using a seven-point scale (Table 1). A description of the development of the ALiEM AIR score, as well as an analysis finding it has moderate to high reliability when used by medical educators to rate online resources, have been previously published [19-20]. Approved resources were assigned to one of two tiers based on the average AIR score achieved according to cutoffs identified using internal data. Those posts that received an average AIR score of 30 or more out of a possible 35 were labeled as “Approved Instructional Resource” while those posts whose average score was less than 30 but 26 or greater were labeled as “Honorable Mention” [3].

**Table 1:** AIR scoring tool AIR: Approved Instructional Resources; EM: Emergency Medicine; EP: Emergency Physicians; BEEM: Best Evidence in Emergency Medicine; EBM: evidenced-based medicine

Tier 1: BEEM Rater Scale	Score	Tier 2: Content accuracy	Score	Tier 3: Educational Utility	Score	Tier 4: EBM	Score	Tier 5: Referenced	Score
Assuming that the results of this article are valid, how much does this article impact on EM clinical practice?		Do you have any concerns about the accuracy of the data presented or conclusions of this article?		Are there useful educational pearls in this article for residents?		Does this article reflect evidence based medicine (EBM)?		Are the authors and literature clearly cited?	
Useless information	1	Yes, many concerns due to many inaccuracies found	1	Not required knowledge for a competent EP	1	Not EBM based, only expert opinion	1	No	1
Not really interesting, not really new, changes nothing	2		2		2		2		2
Interesting and new, but doesn't change practice	3	Yes, a major concern about a few inaccuracies	3	Yes, but there are only a few (1-2) educational pearls that will make the EP a better pracitioner to know or multiple (>=3) educational pearls that are interesting or potentially useful, but rarely required or helpful for the daily practice of an EP.	3	Minimally EBM based	3		3
Interesting and new, has the potential to change practice	4		4		4		4	Yes, authors and general references are listed (but no in-line references)	4
New and important: this would probably change practice for some EPs	5	Minimal concerns, only minor inaccuracies found	5	Yes, there are several (>=3) educational pearls that will make the EP a better practitioner to know, or a few (1-2) every competent EP must know in their practice	5	Mostly EBM based	5		5
New and important: this would change practice for most EPs	6		6		6		6		6
This is a "must know" for EPs	7	No concerns, no inaccuracies found	7	Yes, there are multiple educational pearls that every competent EP must know in their practice	7	Yes exclusively EBM based	7	Yes, authors and in-line references are provided	7
Your Score							

Results

The AIR Series identified two FOAM resources as meeting the criteria for “Approved Instructional Resource” and four posts that scored at the level of “Honorable Mention.” Key educational pearls from each resource are summarized below.

Article 1 (Approved Instructional Resource, AIR score: 30/35)*: *Matthews N. Intra articular lidocaine vs sedation in shoulder reductions. RebelEM. January 30, 2023. https://rebelem.com/intra-articular-lidocaine-vs-procedural-sedation-and-analgesia-for-closed-reduction-of-acute-anterior-shoulder-dislocation/ (Accessed July 27, 2023).

Take-Home Points

This blog post is an evidence-based review of a systematic review and meta-analysis (SRMA) comparing intra-articular lidocaine (IAL) to procedural sedation and analgesia (PSA) for closed reduction of anterior shoulder dislocations.

Summary

Sithamparapillai et al. performed an SRMA of all randomized control trials (RCTs) up to September 2021 that compared IAL to PSA for the closed reduction of anterior shoulder dislocations [21]. While the primary outcome was successful reduction, the authors analyzed multiple secondary outcomes. Ultimately, the authors included 12 RCTs into their analysis consisting of 630 patients. The meta-analysis found no significant difference in the primary outcome of successful reduction. However, there were some significant differences in several of the secondary outcomes. Patients who received IAL experienced fewer adverse events (1.3% vs. 20.8%; RR 0.16; 95% CI 0.07-0.33, I2 = 0, moderate certainty) and had a lower length of stay in the emergency department (ED) (mean difference = − 1.48 h; 95% CI − 2.48 to − 0.47, I2 = 93%, moderate certainty). Patients who underwent PSA had a slightly shorter procedure time (mean difference = 8 min; 95% CI 4.42-11.57, I2 = 97%, moderate certainty) and had higher patient satisfaction scores (70.5% vs. 90.4%; RR 0.80; 95% CI 0.67-0.95, I2 = 71%, moderate certainty). There were several limitations noted, including a small sample size for many of the outcomes, lack of information on patient characteristics, no information on details of reduction techniques or attempts, and moderate to high heterogeneity for almost all of the outcomes that were analyzed, which limits the validity of the meta-analysis component. Ultimately, the blog post authors agreed with the findings of the SRMA that IAL appears to be a safe and effective alternative to PSA in cases of closed reduction of anterior shoulder dislocations.

Article 2 (Approved Instructional Resource, AIR score: 30/35): Jones C. Video laryngoscopy in the ED. emDocs. August 8, 2022*.* http://www.emdocs.net/video-laryngoscopy-in-the-ed-a-review-of-devices-techniques-and-evidence/ (Accessed July 27, 2023).

Take-Home Points

This blog post reviews the different designs available for video laryngoscopy and the different techniques required to use each type. Emergency physicians (EPs) need to be familiar with the differences between standard geometry blades (SGVL) and hyper-angulated blades (HAVL), as both techniques are necessary in the practice of EM.

Summary

There are multiple designs of video laryngoscopes (VLs) that can be used for intubation in the ED. This necessitates the development of expertise in multiple techniques that account for the different variations in design. SGVL and HAVL represent the two basic types of VLs. SGVLs allow for both video and direct views of the airway. HAVLs only have video and do not allow for direct visualization of the airway. Some HAVLs have a channel to facilitate endotracheal tube passage. 

The major advantage of SGVLs is they allow for direct and video views, which means a physician trainee can perform direct laryngoscopy intubation while the supervising physician observes the airway via video. They also allow for the use of airway adjuncts such as the Bougie device. The advantage of HAVLs is that a superior view of the airway is easier to obtain; however, passage of the tube once the view is obtained can be more challenging compared to SGVLs. HAVLs also require the use of a rigid stylet and a more acutely angled endotracheal tube, which limits the use of adjuncts such as a bougie.

Multiple studies show that first-pass success is higher using a VL. However, these studies are generally nonspecific with what device was used and have not compared SGVLs to HAVLs. There is no clear answer to the question of when to use SGVLs versus HAVLs, and that decision will have to be made on a case-by-case basis. As there are several different brands and types of both SGVLs and HAVLs currently available, EPs should ideally learn to be comfortable with all or as many VL models as possible.

Article 3 (Honorable Mention, AIR score: 26): Morgenstern J. Lacerations: does closure technique matter? First 10 in EM. November 28, 2022. https://first10em.com/lacerations-closure-techniques/ (Accessed July 27, 2023).

Take-Home Points

This blog post is an evidence-based review of wound closure techniques. While studies on this topic are of limited quality, techniques for closing simple, uncontaminated, low-tension wounds do not seem to result in major differences in long-term cosmesis. Certain closure techniques (e.g. steri-strips and tissue adhesive) are quicker, cheaper, and less painful than others, although tissue adhesive may be associated with a higher rate of dehiscence when compared to sutures.

Summary

This article discusses the available evidence for wound closure using common techniques in the ED. Techniques reviewed include tissue adhesive, steri-strips, staples, and hair apposition, most of which are compared to sutures. Many of the studies available are small and contain significant bias due to the impossibility of blinding the procedure operator and the patient, as well as the subjectivity in cosmesis as the primary outcome. One additional challenge is the generalizability of skill-based interventions, as the clinician skill level in the studies is often not reported.

There are a small number of RCTs prior to 2000 that look at tissue adhesives, with most reporting use with simple dermal lacerations. They found that tissue adhesives have the benefit of decreased pain scores and procedure time, but do have a slightly increased rate of dehiscence, although this does not appear to significantly affect long-term cosmesis. 

A single RCT for the hair apposition technique compared hair apposition to suturing of scalp lacerations and found hair apposition was quicker, less painful, and preferred to suturing. Ultimately all wounds, whether sutured or closed with hair apposition, were healed at four weeks, with larger scars in the suture group.

A few RCTs comparing tissue adhesive and steri-strips in small, simple lacerations demonstrated no significant difference in cosmesis. There was relative heterogeneity in one systematic review comparing tissue adhesive to adhesive tape in pediatric patients, but it did show a statistically significant benefit of steri-strips compared to adhesives regarding cosmesis. In small studies comparing sutures to steri-strips in small surgical incisions, there were no significant differences noted other than steri-strips were faster to apply. 

Comparing sutures to staples for scalp lacerations, a few small RCTs demonstrated a shorter procedure time for staples with no significant differences in cosmetic outcomes. In two orthopedic surgery meta-analyses looking at sutures versus staples, one found a higher infection rate with staples, and another with poor quality evidence found no difference in infection rate.

Article 4 (Honorable Mention, AIR score: 26): Mulrooney N. Regional nerve blocks module. Don’t Forget the Bubbles. December 7, 2022. https://dontforgetthebubbles.com/regional-nerve-blocks-module/ (Accessed July 27, 2023).

Take-Home Points

This blog post discusses several regional nerve blocks with a focus on pediatric patients that can be used to provide analgesia during the management of injuries to the digits, ear, and hip. It describes the considerations and steps to perform a digital or ulnar nerve block for injuries to the fourth and fifth digits, an auricular block for ear injuries, and a fascia iliaca block for hip injuries.

Summary

This post is designed as an educational module on pediatric regional nerve blocks consisting of an outline, pre-reading assignment, overview, several case-based questions with discussion, a quiz, and take-home points. In the first case, the author describes anesthetic choice, nerve supply, and technique to perform a digital nerve block after the dislocation of the fifth digit at the proximal interphalangeal (PIP) joint. The second case involves using an auricular nerve block to provide analgesia after an earring is stuck in an earlobe and discusses patient positioning, nerve supply, technique, and discharge advice. In the third case, the patient requires a fascia iliaca nerve block after sustaining a hip fracture. The post reviews the equipment and monitoring that is necessary, the anesthetic to use, and the steps to perform the nerve block. Following these initial cases, there are three additional discussion questions posed related to the previous cases. These more advanced discussion topics include using an ulnar nerve block as an alternative to a digital nerve block, the safety of using lidocaine with epinephrine in the digits, and the identification and treatment of local anesthetic systemic toxicity. The author emphasizes the importance of patient positioning, knowing the maximum doses for local anesthetics, appropriate monitoring of the patient, and identifying systemic toxicity when it occurs.

Article 5 (Honorable Mention, AIR score: 27): Victoriano and Avila. Ultrasound Guided Regional Anesthesia for Hip Fractures. emDocs. December 5, 2022. http://www.emdocs.net/ultrasound-guided-regional-anesthesia-for-hip-fractures/. Accessed July 27, 2023.

Take-Home Points

This blog post reviews the use of ultrasound-guided regional anesthesia for hip fractures. Local anesthetic administration for the presurgical treatment of pain in acute hip fractures is preferred to parenteral opiates. There are three commonly used techniques for a fascia iliaca compartment nerve block: the infra-inguinal fascia iliaca nerve block, the suprainguinal fascia iliaca nerve block, and the pericapsular nerve group block (PENG).

Summary 

Alternatives to opiates for the acute management of pain, such as local administration of topical anesthetic, can reduce opiate dependence and side effects. This article outlines treatment options for an acute hip fracture using local anesthetic administration for a fascia iliaca compartment nerve block.

A fascia iliaca compartment nerve block provides analgesia along the femoral nerve, lateral femoral cutaneous nerve, and obturator nerve. It is indicated for hip, knee, and anterior thigh analgesia (e.g. femur fractures, pubic ramus fractures, acetabular fractures, and hip dislocations). There are three techniques of nerve blocks that can be utilized in this setting. 

The first approach is the infra-inguinal fascia iliaca nerve block. This type of nerve block is assisted by using ultrasound to visualize the femoral neurovascular bundle to assist with identifying the correct location for anesthetic injection. The target for injection will be the area just lateral to the femoral nerve, deep to the fascia iliaca, and superficial to the iliacus muscle. The second technique is a suprainguinal fascia iliaca nerve block. This technique may result in a more proximal spread of the anesthetic and successful analgesia. However, a disadvantage of this approach is that it requires visualization of the internal oblique muscle. The final technique is a PENG block. This block is likely superior, due to its more reliable penetration of the obturator nerve, and its motor-sparing effects, and it may be beneficial in acetabular fractures, which are fractures that the fascia iliaca blocks do not reliably anesthetize. The downside is that it can be more technically challenging to perform due to the depth at which the anesthetic needs to be distributed. The blog post contains detailed images as well as step-by-step instructions on how to perform each of the nerve block techniques described in this article.

Article 6 (Honorable Mention, AIR score: 26): Bola A. Ultrasound guided lumbar punctures. Core EM. March 31, 2022. https://coreem.net/core/ultrasound-guided-lumbar-puncture/ (Accessed July 27, 2023).

Take-Home Points

This blog post reviews the use of ultrasound to facilitate the performance of a lumbar puncture (LP). EPs can increase their procedural success in performing LPs by using ultrasound to map necessary landmarks. Ultrasound can aid in identifying the surrounding spinous processes, intervertebral spaces, and subarachnoid space, which can be vital to being successful in otherwise technically difficult patients. 

Summary

The LP is a fundamental procedure for all EPs; however, it can be challenging in certain patients. The procedure involves the insertion of a spinal needle into the intervertebral space in order to collect cerebrospinal fluid for analysis or therapeutic removal. Typically, the targeted intervertebral space is identified using a landmark technique. One study determined that identifying the correct intervertebral space for lumbar puncture via anatomic landmarks was inaccurate in greater than 30% of cases [22].

Ultrasound can be used for mapping the intervertebral spaces, visualizing the depth of the subarachnoid space, as well as identifying other pertinent landmarks for a successful LP. Many studies have examined the use of ultrasound to facilitate LPs and found it to be quick, accurate, and effective.

The first step in ultrasound-guided LP is to choose the appropriate probe. The linear probe is recommended for slender adult and pediatric patients, while the curvilinear probe is recommended for all other patients. The landmarks for the procedure can be determined and marked with the patient in either the sitting or lateral recumbent position. Start with the probe in the transverse orientation and mark the spinous processes on the skin to note the anatomic midline. Turn the probe into a longitudinal orientation and move the probe up or down until you are between two spinous processes. The middle of this distance is the intervertebral space. The LP needle should be inserted at the intersection of the midline and the intervertebral space. The depth for needle insertion can be measured by identifying the subarachnoid space on the longitudinal view. The blog post contains detailed step-by-step instructions as well as annotated images of how to use ultrasound to help guide the LP.

## Conclusions

The ALiEM AIR series curates the highest quality FOAM content on EM subject matter through a reliable methodology of searching the most impactful websites with expert review using a standardized scoring instrument to provide learners with a vetted resource for learning. Educators also benefit from this series through its ease of use and incorporation into EM training curricula. This review was limited by the inclusion of resources from the top 50 sites on the SMi published from January 2022 to January 2023. Therefore, it is possible that resources that were otherwise of sufficient quality and relevance failed to be reviewed. In this review, we summarize the key educational pearls from the six FOAM resources that were curated by the AIR series and earned the title of either “Approved Instructional Resource” or “Honorable Mention” in January 2023, representing the highest quality FOAM content currently available on the topic of EM procedures.

## References

[1] Pearson D, Cooney R, Bond MC (2015). Recommendations from the Council of Residency Directors (CORD) Social Media Committee on the role of social media in residency education and strategies on implementation. West J Emerg Med.

[2] Cadogan M, Thoma B, Chan TM, Lin M (2014). Free Open Access Meducation (FOAM): the rise of emergency medicine and critical care blogs and podcasts (2002-2013). Emerg Med J.

[3] Lin M, Joshi N, Grock A (2016). Approved Instructional Resources Series: a national initiative to identify quality emergency medicine blog and podcast content for resident education. J Grad Med Educ.

[4] Zaver F, Hansen M, Leibner E, Little A, Lin M (2016). Blog and podcast watch: pediatric emergency medicine. West J Emerg Med.

[5] Grock A, Joshi N, Swaminathan A, Rezaie S, Gaafary C, Lin M (2016). Blog and podcast watch: neurologic emergencies. West J Emerg Med.

[6] Grock A, Morley EJ, Roppolo L, Khadpe J, Ankel F, Lin M (2017). Blog and podcast watch: cutaneous emergencies. West J Emerg Med.

[7] Grock A, Rezaie S, Swaminathan A, Min A, Shah KH, Lin M (2017). Blog and podcast watch: orthopedic emergencies. West J Emerg Med.

[8] Zaver F, Craddick M, Sanford A, Sefa N, Hughes G, Lin M (2017). ALiEM blog and podcast watch: toxicology. West J Emerg Med.

[9] Joshi N, Morley EJ, Taira T, Branzetti J, Grock A (2017). ALiEM blog and podcast watch: procedures in emergency medicine. West J Emerg Med.

[10] Roppolo L, Gaafary C, Khadpe J, Shah K, Grock A (2018). Academic Life in Emergency Medicine (ALiEM) blog and podcast watch: infectious disease emergencies. Cureus.

[11] Min AA, Morley EJ, Rezaie SR, Fox SM, Grock A (2018). Academic life in emergency medicine blog and podcast watch: respiratory emergencies. Cureus.

[12] Grock A, Wheaton N, Roppolo L, Gaafary C (2018). Academic life in emergency medicine blog and podcast watch: toxicologic emergencies. Cureus.

[13] Min AA, Jordan J, Swaminathan A, Hennings J, Grock A (2018). Academic Life in Emergency Medicine (ALiEM) blog and podcast watch: renal and genitourinary emergencies. Cureus.

[14] Ediger D, Sumpter R, Bridwell RE, Belcher CN (2020). Academic Life in Emergency Medicine (ALiEM) blog and podcast watch: infectious diseases. Cureus.

[15] Thoma B, Sanders JL, Lin M, Paterson QS, Steeg J, Chan TM (2015). The social media index: measuring the impact of emergency medicine and critical care websites. West J Emerg Med.

[16] Thoma B, Chan TM, Kapur P (2018). The social media index as an indicator of quality for emergency medicine blogs: A METRIQ study. Ann Emerg Med.

[17] Lin M, Phipps M, Chan TM (2023). Digital impact factor: a quality index for educational blogs and podcasts in emergency medicine and critical care. Ann Emerg Med.

[18] Beeson MS, Ankel F, Bhat R (2020). The 2019 model of the clinical practice of emergency medicine. J Emerg Med.

[19] Grock A, Jordan J, Zaver F (2021). The revised Approved Instructional Resources score: An improved quality evaluation tool for online educational resources. AEM Educ Train.

[20] Chan TM, Grock A, Paddock M, Kulasegaram K, Yarris LM, Lin M (2016). Examining Reliability and Validity of an Online Score (ALiEM AIR) for rating Free Open Access Medical Education Resources. Ann Emerg Med.

[21] Sithamparapillai A, Grewal K, Thompson C, Walsh C, McLeod S (2022). Intra-articular lidocaine versus intravenous sedation for closed reduction of acute anterior shoulder dislocation in the emergency department: a systematic review and meta-analysis. CJEM.

[22] Duniec L, Nowakowski P, Kosson D, Łazowski T (2013). Anatomical landmarks based assessment of intravertebral space level for lumbar puncture is misleading in more than 30%. Anaesthesiol Intensive Ther.

